# Localized Multidrug-Resistant *Morganella morganii* Urinary Tract Infection in a Dapagliflozin-Treated Man with Bladder Outlet Obstruction and Incomplete Ureteral Duplication: A Case Report

**DOI:** 10.3390/reports9030292

**Published:** 2026-09-01

**Authors:** Camelia Nicolae, Madalina Andreea Munteanu, Razvan Petca, Stefan Alexandru Rascu, Razvan Alexandru Danau

**Affiliations:** 1 Internal Medicine and Cardiology Department, Carol Davila University of Medicine and Pharmacy, 020021 Bucharest, Romania; 2 Cardiology and Urology Department, Clinical Hospital “Prof. Dr. Th. Burghele”, 050659 Bucharest, Romaniarazvan.danau@umfcd.ro (R.A.D.); 3 Urology Department, Carol Davila University of Medicine and Pharmacy, 020021 Bucharest, Romania

**Keywords:** SGLT2 inhibitor, *Morganella morganii*, benign prostatic hyperplasia, post-void residual, urinary tract infection

## Abstract

**Background and Clinical Significance:** Sodium–glucose cotransporter-2 (SGLT2) inhibitors cause persistent glucosuria, which has been hypothesized to facilitate urinary tract infection (UTI) when urinary stasis or anatomic variants coexist; causality, however, remains unproven and almost certainly multifactorial. **Case Presentation:** A 72-year-old man treated with dapagliflozin 10 mg/day for 4 months presented with 3 days of dysuria and nocturia, without fever or systemic signs. One month before SGLT2 inhibitor initiation, urological work-up had been unremarkable: prostate ~50 cm^3^, post-void residual (PVR) 0 mL, and a negative midstream culture. At presentation we obtained a midstream clean-catch specimen before antibiotics. Dipstick showed glucose 2+ (~100 mg/dL, as expected with SGLT2 inhibition), leukocyte esterase 3+ and nitrites negative; microscopy revealed 45 leukocytes/high-power field with pronounced bacteriuria. Quantitative culture grew *Morganella morganii* at 1 × 10^5^ CFU/mL in pure culture. Antimicrobial susceptibility (VITEK^®^ 2 Compact, EUCAST v14.0, 2024) demonstrated resistance to ampicillin, amoxicillin–clavulanate, ampicillin–sulbactam, ceftriaxone, ceftazidime, gentamicin and trimethoprim–sulfamethoxazole, with susceptibility (S) retained only to piperacillin–tazobactam, levofloxacin/norfloxacin, cefepime and carbapenems (ertapenem, meropenem). Imaging revealed a right duplicated ureter with distal fusion (single bladder insertion, no dilatation or obstruction) and benign prostatic hyperplasia with PVR ~100 mL; renal function was preserved, two blood culture sets (pre-antibiotic) were negative, and repeat urine culture on day 7 was sterile. Management/outcome: The episode was classified as a localized (cystitis-range) UTI per European Association of Urology (EAU) criteria (no fever, flank pain or bacteremia). Meropenem 1 g intravenously every 8 h (3 g/day) was given for 10 days after urology/infectious-disease review. Dapagliflozin was held at presentation and not restarted during the 3-month follow-up period; reintroduction was planned only after full urological reassessment with cardiology input. Transurethral resection of the prostate (TUR-P) was performed 18 days after completing antibiotics (28 days after presentation) for bladder outlet obstruction refractory to tamsulosin 0.4 mg daily, with complete resolution of symptoms, normalization of inflammatory markers and PVR 0 mL. No recurrence occurred during 3 months of follow-up after TUR-P. **Conclusions:** This single case illustrates a temporal association—not proven causation—between SGLT2-related glucosuria, incomplete emptying and a non-obstructive duplicated ureter that likely created a permissive milieu for opportunistic Morganella UTI. The narrative is hypothesis-generating; urinary stasis was the dominant modifiable factor. Culture-guided therapy, individualized decisions on SGLT2 continuation, and definitive correction of outlet obstruction are the practical takeaways.

## 1. Introduction and Clinical Significance

Sodium–glucose cotransporter-2 (SGLT2) inhibitors have reshaped cardiometabolic care by combining glucose lowering with meaningful benefits in heart failure and chronic kidney disease in people with and without diabetes. These agents block SGLT2 in the early proximal tubule, thereby lowering the threshold for urinary glucose excretion and producing sustained glucosuria with accompanying osmotic diuresis and natriuresis. The downstream hemodynamic and metabolic effects—lower intraglomerular pressure, modest weight and blood pressure reduction, and improved heart failure outcomes—have moved these drugs beyond endocrinology into cardiology and nephrology. As use expands, genitourinary adverse events are encountered more often and require individualized, patient-level risk assessment [1].

Genitourinary infections are among the most frequently discussed safety signals with SGLT2 inhibitors. Mechanistically, glucose-rich urine can raise urinary osmolarity and provide a fermentable substrate that may favor microbial proliferation when host defenses or washout are impaired. In patients with robust urothelial immunity and efficient bladder emptying, this perturbation by itself often does not translate into symptomatic infection. However, when urine flow is impeded or residual volumes persist, bacterial dwell time increases, and the likelihood of adherence, biofilm formation, and ascending colonization rises. Such conditions occur in common clinical scenarios—benign prostatic hyperplasia (BPH) with incomplete emptying, neurogenic bladder, instrumentation, indwelling catheters, and structural anomalies of the collecting system or ureters [2]. Importantly, large trial and meta-analytic data indicate that this mechanistic concern does not translate into a large increase in overall UTI risk at the population level, underscoring the need for individualized assessment rather than routine drug discontinuation [1,2,3,4].

In infections linked to stasis or other complicating factors, the microbiology shifts away from the *Escherichia coli* predominance seen in uncomplicated community-acquired cystitis. Organisms that behave opportunistically—*Proteus* spp., *Klebsiella* spp. and, far less often, *Morganella morganii*—are isolated more frequently when host defenses or urinary washout are impaired by anatomical or functional abnormalities, instrumentation, or metabolic/immune stress. *Morganella morganii*, a gut-resident Enterobacterales member, is intrinsically non-susceptible to many penicillins and early-generation cephalosporins and readily acquires additional resistance determinants, which narrows empirical choices and makes susceptibility-guided therapy essential. Culture results therefore need to be interpreted together with a search for correctable urological contributors, not as isolated microbiology [3,4].

These intersecting themes—SGLT2-related glucosuria, BPH-related incomplete bladder emptying, and a non-obstructive duplicated ureter—frame the case we report: a 72-year-old man on dapagliflozin who developed a localized UTI due to multidrug-resistant *Morganella morganii*. We detail the diagnostic reasoning, antimicrobial selection using EUCAST standards, and the rationale for individualized temporary interruption of SGLT2 therapy alongside definitive management of bladder outlet obstruction with transurethral resection of the prostate (TUR-P). We situate the case within contemporary guideline recommendations and comparative evidence, emphasizing a hypothesis-generating temporal association rather than proven causality and highlighting a practical approach to preserving the cardiorenal benefits of SGLT2 inhibitors while mitigating infection risk in patients with anatomical or functional urinary tract vulnerabilities [2,5,6].

## 2. Case Presentation

A 72-year-old Caucasian man presented to the emergency department with a 3-day history of fatigue, dysuria, and increased nocturnal urination, without fever, flank pain, dysuria-related fever, or costovertebral angle tenderness. His past medical history included coronary artery disease, heart failure with preserved ejection fraction (NYHA class I), hypertension, obesity, and benign prostatic hyperplasia (BPH) with lower urinary tract symptoms treated with tamsulosin 0.4 mg daily for the preceding 6 months (started 2 months before dapagliflozin). Four months prior to the current episode, he had begun therapy with dapagliflozin 10 mg once daily for heart failure with preserved ejection fraction. One month before initiation of dapagliflozin, the patient underwent complete urological evaluation: prostate-specific antigen (PSA) was 2.24 ng/mL; midstream urine culture was negative (<10^3^ CFU/mL); digital rectal examination showed a non-tender, enlarged but flat prostate without fluctuance or tenderness suggestive of prostatitis; renal ultrasound demonstrated normal renal morphology, no collecting system dilatation and no calculi; prostate volume was ~50 cm^3^; and PVR urine was 0 mL (complete emptying). No prostatitis was suspected clinically, and no prior UTIs had been documented.

Following that evaluation, the patient was deemed a suitable candidate for dapagliflozin given the benefit in heart failure with preserved ejection fraction. His comorbidities were managed with daily low-dose aspirin 75 mg, fixed-dose amlodipine 5 mg/perindopril 5 mg/indapamide 1.25 mg for hypertension, rosuvastatin 20 mg for dyslipidemia, and tamsulosin 0.4 mg daily for BPH, which was continued unchanged until presentation. He had no history of tobacco or alcohol use, and family history revealed hypertension and cardiovascular disease in first-degree relatives.

On examination, body mass index was 32.5 kg/m^2^. Vital signs were temperature 36.0 °C, heart rate 84 bpm, blood pressure 146/86 mmHg, respiratory rate 17/min, SpO_2_ 97% on room air. Physical examination revealed no suprapubic tenderness, no costovertebral angle tenderness, and no urethral discharge. Electrocardiogram showed sinus rhythm at 90 bpm without ST-segment or repolarization changes. Point-of-care transthoracic echocardiography showed a non-dilated, non-hypertrophied left ventricle with preserved ejection fraction, no regional wall motion abnormalities, grade I diastolic dysfunction, mild mitral, aortic and tricuspid regurgitation, no right-sided enlargement, no pulmonary hypertension, and no pericardial effusion.

### 2.1. Diagnostic Assessment

Urine sampling and microbiology: A midstream clean-catch specimen was obtained after standard periurethral cleansing, before antibiotic initiation. Dipstick urinalysis showed glucose positive (2+, ~100 mg/dL) consistent with expected SGLT2 inhibitor–induced glucosuria, leukocyte esterase positive (3+), nitrite negative, pH 6.0, and no ketones. Microscopy revealed 45 leukocytes/high-power field (HPF), 8 erythrocytes/HPF, and pronounced bacteriuria without squamous epithelial contamination. Quantitative culture on chromogenic and blood agar (incubated 18–24 h at 35 ± 2 °C) grew *Morganella morganii* at 1 × 10^5^ colony-forming units (CFU)/mL in pure culture. Repeat midstream culture on day 7 of therapy was sterile (<10^3^ CFU/mL). Two sets of blood cultures drawn at presentation (before antibiotics) remained negative after 5 days. Prostatitis was considered clinically unlikely: the patient was afebrile and denied perineal pain; digital rectal examination showed no prostatic tenderness, fluctuance or warmth, PSA was 2.24 ng/mL (stable), urinalysis without clumping, and no prostate massage or expressed secretion sampling was indicated. Antimicrobial susceptibility testing (AST) was performed with the VITEK^®^ 2 Compact automated system (bioMérieux) and interpreted according to EUCAST v14.0 (2024) breakpoints. Zone diameters/MICs where available are presented with categorical interpretation using EUCAST terminology (Susceptible, S; Susceptible, increased exposure, I; Resistant, R). The isolate showed multidrug resistance with preserved susceptibility only to piperacillin–tazobactam (MIC 8 mg/L, S), norfloxacin (S, zone 22 mm), levofloxacin (MIC 0.5 mg/L, S), cefepime (MIC 2 mg/L, S), ertapenem (MIC 0.5 mg/L, S) and meropenem (MIC 1 mg/L, S), as well as resistance to ampicillin, amoxicillin–clavulanate, ampicillin–sulbactam, ceftriaxone, ceftazidime, gentamicin, and trimethoprim–sulfamethoxazole (Table 1). The retained susceptibility to cefepime despite intrinsic resistance to many beta-lactams was confirmed in vitro by the automated dilution method; no molecular testing for AmpC/ESBL genes was performed, but phenotypic testing showed no extended-spectrum beta-lactamase phenotype, and cefepime susceptibility was interpreted strictly per EUCAST criteria for Enterobacterales. We note that in vitro susceptibility does not guarantee clinical efficacy and was considered alongside the patient’s risk factors and disease severity. Norfloxacin susceptibility is reported per EUCAST only for uncomplicated UTI and was not considered suitable for this complicated, retention-associated case (see management rationale).

Laboratory testing on admission showed mildly elevated inflammatory markers with preserved renal function (Table 2): white blood cells 10.8 × 10^9^/L (ref 4.0–10.0), neutrophils 7.2 × 10^9^/L, C-reactive protein 28 mg/L (ref < 5), procalcitonin 0.18 ng/mL (ref < 0.05), creatinine 88 µmol/L (1.00 mg/dL), estimated glomerular filtration rate 78 mL/min/1.73 m^2^, blood urea nitrogen 6.1 mmol/L, glucose 5.9 mmol/L (106 mg/dL), HbA1c 5.7%, and urinalysis as above. Pelvic and abdominal ultrasonography confirmed prostatic enlargement (volume ~50 cm^3^) and demonstrated incomplete bladder emptying with a post-void residual (PVR) volume of ~100 mL measured by bladder scan immediately after voiding. In contrast to the pre-dapagliflozin assessment 1 month before SGLT2 inhibitor initiation (PVR 0 mL), this represented new/worsening retention despite continued alpha-blocker therapy. No hydronephrosis or upper-tract dilatation was seen on ultrasound.

**Figure 1:**
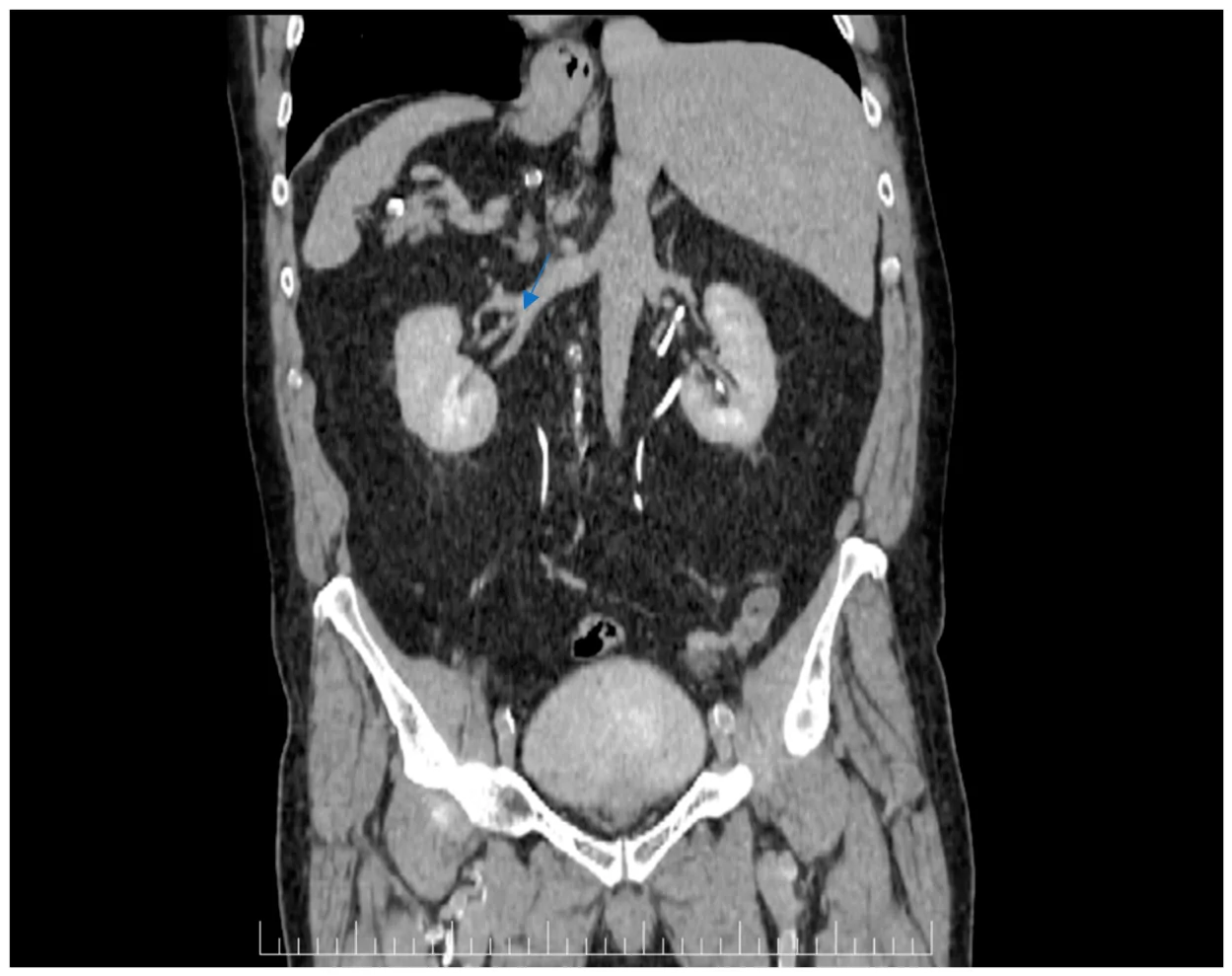
Contrast-enhanced computed tomography (CT) image of the abdomen and pelvis in the coronal plane, showing a right-sided duplicated pyeloureteral system with distal fusion near the vesicoureteral junction resulting in a single ureteral insertion into the bladder (arrows). No hydronephrosis, ureteral dilatation, ectopic insertion, or obstructive uropathy was observed.

Contrast-enhanced CT of the abdomen and pelvis identified a right-sided duplicated pyeloureteral system characterized by apparent distal fusion of both ureters near the vesicoureteral junction, resulting in a single ureteral insertion into the bladder (Figure 1). Importantly, there was no ureteral dilatation, ectopic insertion, hydronephrosis, or evidence of obstructive uropathy or vesicoureteral reflux on imaging. This congenital variant had not previously been associated with infections or lower urinary tract symptoms, and we interpret it as a non-obstructive anatomical variant that by itself does not explain infection but may modestly increase susceptibility when combined with functional stasis.

### 2.2. Management

Considering the patient’s lower urinary tract symptoms without fever, flank pain, costovertebral angle tenderness, or bacteremia and imaging ruling out upper-tract involvement, the infection was categorized as a localized UTI (cystitis-range) per the EAU framework differentiating localized from febrile/systemic UTI [3]. Antibiotic treatment was selected based on the quantitative urine culture and EUCAST v14.0 susceptibility, aiming to target *Morganella morganii* while balancing efficacy and stewardship. Meropenem 1 g intravenously every 8 h (3 g/day) for 10 days was initiated on the day of presentation, simultaneously with temporary discontinuation of dapagliflozin, following joint urology and infectious-disease consultation. The rationale for meropenem—despite in vitro susceptibility to levofloxacin, cefepime, and piperacillin–tazobactam—included: (i) the multidrug-resistant phenotype with resistance to multiple beta-lactams and aminoglycosides, (ii) the need for reliable bactericidal activity in an older adult with ~100 mL PVR and incomplete emptying where fluoroquinolone tissue variability and stewardship concerns for fluoroquinolones were considered, (iii) cefepime susceptibility being phenotypically unusual for Morganella and not confirmed by molecular testing, prompting caution for definitive therapy in a complicated host, and (iv) local stewardship guidance favoring a short, definitive carbapenem course with documented clearance rather than prolonged broad-spectrum exposure. Piperacillin–tazobactam was considered but de-prioritized given recent data on inferior outcomes in some AmpC-producing Enterobacterales with high-inoculum infection and the desire for once-stable outpatient management without extended infusion. We acknowledge that carbapenem use contradicts a simplistic narrowest spectrum narrative; stewardship here was pursued by a finite 10-day course, no additional agents, documented microbiological clearance (repeat sterile culture day 7), and avoidance of prophylactic continuation. Supportive care included adequate hydration and symptomatic treatment; no urinary catheter was placed. Dapagliflozin discontinuation was an individualized clinical decision based on ongoing bacteriuria, glucosuria, and urinary retention—not a blanket guideline mandate. Current expert consensus [5,6] does not recommend routine discontinuation of SGLT2 inhibitors for mild-to-moderate genitourinary infections but suggests an individualized pause in the presence of severe, persistent, or complicated infection or marked stasis; we followed that individualized approach. The patient was counseled that reintroduction would be considered only after infection resolution and definitive urological correction of stasis, with cardiology input on risk–benefit.

Although BPH had been treated with tamsulosin 0.4 mg daily for 6 months (started 2 months before dapagliflozin), reassessment during hospitalization revealed persistent and worsening lower urinary tract symptoms (International Prostate Symptom Score 18, moderate–severe) and PVR ~100 mL, indicating inadequate bladder emptying despite optimized alpha-blockade [7,8]. No anticholinergics or 5-alpha-reductase inhibitors were used. Per EAU Guidelines on non-neurogenic male lower urinary tract symptoms, surgical evaluation is warranted for persistent symptoms and significant PVR after adequate medical therapy [7]. After confirming a sterile repeat urine culture and normalization of CRP (4.2 mg/L), the patient proceeded to transurethral resection of the prostate (TUR-P).

Timeline of care: Day 0 (presentation): Midstream culture + blood cultures, meropenem 1 g q8h started, dapagliflozin held. Day 3: Symptomatic improvement (dysuria resolving), afebrile. Day 7: Repeat urine culture sterile, CRP 11 mg/L. Day 10: Meropenem completed (total 10 days). Day 28 (18 days after completing antibiotics): TUR-P performed (uncomplicated, catheter removed on postoperative day 2). Week 6: PVR 0 mL, IPSS 7 (mild). During 3 months of follow-up after TUR-P (to ~4 months after presentation), the patient remained asymptomatic, with a negative repeat dipstick, no UTI recurrence, stable creatinine, and PVR that remained at 0 mL. Dapagliflozin was not restarted during this 3-month follow-up period; the patient remained on aspirin, amlodipine/perindopril/indapamide, rosuvastatin, and tamsulosin was discontinued post-TUR-P per urology. Reintroduction of an SGLT2 inhibitor is planned only after a shared cardiology–urology decision and was intentionally deferred until definitive outlet correction and a recurrence-free interval, underscoring the need for individualized benefit–risk reassessment in patients with urological comorbidities [5,6].

Overall, management prioritized rapid microbiological clearance, temporary individualized interruption of the SGLT2 inhibitor, and definitive correction of bladder outlet obstruction to reduce recurrence risk.

## 3. Discussion

Developmental anomalies of the urinary tract are recognized to increase susceptibility to infection, most clearly when they lead to urinary stasis, vesicoureteral reflux or incomplete obstruction [8,9,10,11]. Ureteral duplication, one of the commonest such variants, is typically asymptomatic and often discovered incidentally. Studies of adults with duplicated systems show that most remain free of symptomatic infection unless coexistent factors that impair emptying or drainage are present [12].

In this case, contrast-enhanced CT identified an incidental right-sided duplicated pyeloureteral system with apparent distal fusion near the vesicoureteral junction and a single bladder insertion. The absence of hydronephrosis, obstructive features, or ectopic insertion suggests a non-obstructive variant. Current evidence suggests that analogous non-obstructive duplicated arrangements do not intrinsically predispose to recurrent UTI unless accompanied by secondary factors such as bladder outlet obstruction or functional urine retention [12,13]. We therefore do not attribute causation to the duplication alone; rather, we view it as a minor anatomical cofactor that may modestly reduce washout efficiency when retention coexists.

The case-based literature illustrates that the spectrum of SGLT2 inhibitor-associated UTI is often driven by host factors rather than the drug alone.

Similarly, *Morganella morganii* infection is best understood as opportunistic rather than primarily uropathogenic. A 2025 pediatric case of Morganella UTI in a child with congenital hydronephrosis and multiple adult reports in settings of stasis, catheterization, or immunocompromise reinforce its behavior as a pathogen that colonizes when host defenses or flow are compromised rather than as a classic community-acquired cystitis organism [4,13]. In vitro, Morganella carries intrinsic resistance to ampicillin, amoxicillin–clavulanate and many cephalosporins via chromosomal AmpC, which makes the retained susceptibility to cefepime in our isolate noteworthy. This susceptibility pattern represents a confirmed in vitro finding according to EUCAST v14.0, without molecular characterization. Although cefepime susceptibility may be observed in Morganella morganii isolates lacking derepressed AmpC overexpression or concurrent ESBL carriage, the absence of PCR confirmation for blaDHA, blaCMY, or ESBL determinants warrants cautious clinical correlation. This uncertainty guided our preference for meropenem as definitive therapy in a patient with significant urinary stasis.

The role of SGLT2 inhibitors requires careful framing. In non-diabetic individuals, SGLT2 inhibition produces sustained glucosuria—a biologically plausible substrate for Enterobacterales growth. The systematic review and meta-analysis by Puckrin et al. [14] (86 trials, ~51,000 participants) found no significant increase in overall UTI risk versus placebo (RR 1.03, 95% CI 0.96–1.11) or versus active comparators. Another population-based cohort reported a modestly higher rate of severe UTI (pyelonephritis, urosepsis, hospitalization) versus DPP-4 inhibitors/GLP-1 agonists, but absolute rates remained low [15]. In our patient, the principal modifiable risk factor was BPH-related incomplete emptying with a persistent PVR of ~100 mL. EAU guidance indicates that PVR volumes >50–100 mL persisting despite adequate medical therapy substantially increases UTI risk [7,11]. Moreover, series in men with chronic urinary retention report a higher prevalence of opportunistic Gram-negative organisms—notably *Proteus* spp. and *Klebsiella* spp. and less frequently *Morganella* spp.—than is observed in uncomplicated community-acquired Escherichia coli cystitis [4].

Collectively, this case suggests a multifactorial, permissive interplay rather than proven synergistic causation. A non-obstructive duplicated ureter, inadequate bladder emptying due to BPH, and pharmacologically induced glucosuria may together have created an environment that permitted colonization and symptomatic infection by an opportunistic, multidrug-resistant *Morganella morganii*. No single factor alone—BPH with PVR 100 mL, a non-obstructive duplication, or glucosuria—would usually be sufficient to explain this specific pathogen, but their coincidence likely impaired washout and host defense. Urinary stasis remains the most actionable contributor.

The relationship between SGLT2 inhibitors and UTIs remains actively debated. Large cardiovascular and renal outcome trials (DAPA-HF, EMPEROR-Preserved, DAPA-CKD) [16] enrolling substantial non-diabetic populations did not demonstrate a significant excess of severe UTIs, while observational cohorts suggest that UTIs may be more prevalent in vulnerable subgroups with retention, anatomical variants, or obesity [14,15,16,17]. Another case reported a 39-year-old woman with type 2 diabetes who developed recurrent, febrile UTI with *Escherichia coli* (1 × 10^5^ CFU/mL) and pyelonephritis-like features 2 months after starting empagliflozin 10 mg; symptoms resolved after drug withdrawal and antibiotic therapy, without claiming mixed Klebsiella/Citrobacter growth [18]. Other small series and isolated reports have described mixed growth (e.g., Klebsiella, Enterococcus, Citrobacter) in stasis-prone hosts, but these are heterogeneous and cannot establish incidence or causality. Taken together, such cases are best viewed as hypothesis-generating: glucosuria may act as a permissive substrate only when urinary stasis or other predisposing factors coexist.

This case aligns with the latter pattern: dapagliflozin-associated glucosuria may have acted as a permissive cofactor—not a direct cause—alongside demonstrable urological stasis. The clinical implication is not routine SGLT2 inhibitor discontinuation but individualized risk assessment: evaluating urinary history, PVR, and anatomical factors before and during therapy, pursuing early culture-guided treatment when symptoms arise, and considering temporary individualized interruption only for severe, persistent, or complicated genitourinary infection per expert consensus [5,6,16,17]. This case also illustrates why correcting outlet obstruction is central to prevention, potentially allowing future reassessment of SGLT2 inhibitor reinitiation under shared decision-making.

## 4. Conclusions

This single case describes a localized, non-bacteremic, afebrile urinary tract infection caused by multidrug-resistant Morganella morganii in a 72-year-old man receiving dapagliflozin 10 mg daily, occurring in the context of bladder outlet obstruction with a persistent post-void residual volume of approximately 100 mL and an incidental, non-obstructive ureteral duplication. The relationship is best characterized as a temporal, hypothesis-generating association rather than a causal link: SGLT2 inhibitor-associated glucosuria may have contributed as a permissive factor, whereas urinary stasis constituted the principal modifiable determinant. For clinical practice, we suggest heightened vigilance for atypical or opportunistic uropathogens when UTI occurs in SGLT2 inhibitor–treated patients with urinary retention or genitourinary anatomical variants, prompt urological evaluation even in the absence of overt obstruction, and individualized risk–benefit assessment regarding continuation or temporary interruption of SGLT2 inhibitor therapy—rather than routine, guideline-mandated discontinuation—while prioritizing definitive correction of urinary stasis to preserve the established cardiorenal benefits of this drug class where feasible.

## Figures and Tables

**Table 1:** Antimicrobial susceptibility profile of *Morganella morganii* isolated from urine culture.

Germ Identified:	*Morganella morganii*
Ampicillin	Resistant (R)—MIC > 16 mg/L
Amoxicillin–clavulanic acid	Resistant (R)—MIC 32 mg/L
Piperacillin–tazobactam	Susceptible (S)—MIC 8 mg/L
Ampicillin–sulbactam	Resistant (R)
Ceftriaxone	Resistant (R)—MIC 4 mg/L
Ceftazidime	Resistant (R)—MIC 8 mg/L
Gentamicin	Resistant (R)—MIC > 8 mg/L
Norfloxacin	Susceptible (S)—zone 22 mm *
Levofloxacin	Susceptible (S)—MIC 0.5 mg/L
Trimethoprim–sulfamethoxazole	Resistant (R)
Ertapenem	Susceptible (S)—MIC 0.5 mg/L
Meropenem	Susceptible (S)—MIC 1 mg/L
Cefepime	Susceptible (S)—MIC 2 mg/L

Method: VITEK^®^ 2 Compact (bioMérieux), 18–24 h at 35 ± 2 °C, interpreted per EUCAST v14.0 (2024). EUCAST terminology: S = Susceptible, R = Resistant. * Norfloxacin per EUCAST only for uncomplicated cystitis; not suitable for this complicated retention-associated UTI.

**Table 2:** Routine laboratory tests at admission and microbiological results (pre- and post-treatment). EUCAST v14.0.

Parameter	Value (Reference Range)
White blood cells	10.8 ×10^9^/L (4.0–10.0)
Neutrophils	7.2 ×10^9^/L (1.8–7.0)
C-reactive protein	28 mg/L (<5) → 4.2 mg/L at day 10
Procalcitonin	0.18 ng/mL (<0.05)
Creatinine	88 µmol/L (62–106)–1.00 mg/dL
eGFR (CKD-EPI)	78 mL/min/1.73 m^2^ (>60)
BUN	6.1 mmol/L (2.9–8.2)
Glucose (random)	5.9 mmol/L (3.9–5.6)–106 mg/dL
HbA1c	5.7% (<5.7)
PSA (1 month pre-dapagliflozin)	2.24 ng/mL (<4.0)
Urinalysis—leukocyte esterase	3+ positive
Urinalysis—nitrite	Negative
Urinalysis—glucose	2+ positive (~100 mg/dL) *
Urinalysis—microscopy leukocytes	45/HPF (<5), 8 erythrocytes/HPF
Urinalysis—squamous epithelial cells	Few (no contamination)
Blood cultures (2 sets, pre-antibiotic)	Negative after 5 days
Repeat urine culture (day 7)	Sterile (<10^3^ CFU/mL)

* Dipstick glucose reflects expected SGLT2 inhibitor–induced glucosuria; quantitative urinary glucose excretion not measured. Midstream clean-catch specimen, 1 × 10^5^ CFU/mL pure culture. No catheter. Prostatitis clinically excluded (afebrile, no prostatic tenderness, no perineal pain).

## Data Availability

All relevant clinical data supporting this case are contained within the manuscript and tables. Additional anonymized details are available from the corresponding author upon reasonable request, subject to privacy restrictions.

## Abbreviations

The following abbreviations are used in this manuscript:

SGLT2	Sodium–glucose cotransporter-2
UTI	Urinary tract infection
BPH	Benign prostatic hyperplasia
PVR	Post-void residual (urine)
TUR-P	Transurethral resection of the prostate
EAU	European Association of Urology
EUCAST	European Committee on Antimicrobial Susceptibility Testing
CFU	Colony-forming units
MIC	Minimum inhibitory concentration
AST	Antimicrobial susceptibility testing
CRP	C-reactive protein
eGFR	Estimated glomerular filtration rate
PSA	Prostate-specific antigen
HPF	High-power field
BMI	Body mass index
IPSS	International Prostate Symptom Score
